# Synthesis of CdSe and CdSe/ZnS Quantum Dots with Tunable Crystal Structure and Photoluminescent Properties

**DOI:** 10.3390/nano12172969

**Published:** 2022-08-27

**Authors:** Jingling Li, Haixin Zheng, Ziming Zheng, Haibo Rong, Zhidong Zeng, Hui Zeng

**Affiliations:** 1 School of Materials Science and Hydrogen Energy, Foshan University, Foshan 528000, China; 2 Guangdong Key Laboratory for Hydrogen Energy Technologies, Foshan University, Foshan 528000, China; 3 School of Light Industry and Materials, Guangdong Polytechnic, Foshan 528041, China

**Keywords:** quantum dot, CdSe, crystal structure control, photoluminescence

## Abstract

Mastery over the structure of nanocrystals is a powerful tool for the control of their fluorescence properties and to broaden the range of their applications. In this work, the crystalline structure of CdSe can be tuned by the precursor concentration and the dosage of tributyl phosphine, which is verified by XRD, photoluminescence and UV-vis spectra, TEM observations, and time-correlated single photon counting (TCSPC) technology. Using a TBP-assisted thermal-cycling technique coupled with the single precursor method, core–shell QDs with different shell thicknesses were then prepared. The addition of TBP improves the isotropic growth of the shell, resulting in a high QY value, up to 91.4%, and a single-channel decay characteristic of CdSe/ZnS quantum dots. This work not only provides a facile synthesis route to precisely control the core–shell structures and fluorescence properties of CdSe nanocrystals but also builds a link between ligand chemistry and crystal growth theory.

## 1. Introduction

Colloidal semiconductor nanocrystals (NCs) have unique photophysical properties, such as size-tunable symmetric emission, superior light absorbance, and high photoluminescence quantum yield [1,2,3]. They have shown good application prospects in optoelectronic devices, immunoassays, and bio-imaging [4,5,6,7,8]. In 1993, Bawendi’s group introduced a high-temperature synthesis method in organic solvents, which was a significant milestone in the preparation of monodisperse NCs [9]. It described the synthesis of cadmium chalcogenides (CdE, E = S, Se, and Te), and since then, CdSe quantum dots (QDs) have served as the main subject of a great number of studies. In most studies, tailoring the optical and electrical properties of NCs is the primary task because optoelectronics applications usually have a high demand for color precision [10,11]. Minimizing the size variability during the synthesis of QDs is desirable. It has been proven that the type of precursors, surfactants, molarity, and growth temperature influence the manipulability of the size and shape and, hence, the optical and electrical properties of NCs [12,13,14]. The last decade has witnessed the success of protocols that allow for control of the size and shape of NCs, creating NCs with accurate and peculiar morphologies [15].

In recent years, the photoelectric properties of QDs have been improving, which creates an opportunity for QDs to revolutionize the field of laser-based optoelectronics [16,17,18]. However, there is high demand for NCs, specifically those with monotypic radiative recombination besides the high quantum yield [19]. To achieve single radiative decay in its intrinsic channel, non-radiative recombination should be eliminated. In general, the non-radiative transition is associated with levels from intrinsic or surface defects. Therefore, the control of crystal structure is the key to achieving these ideal fluorescence properties. Different kinds of CdSe NCs, including both the wurtzite and the zinc blende structures, have been studied. Theoretical calculations revealed that the energy difference between zinc blende and wurtzite structures is only 0.14 kJ/mol per unit [20]. Therefore, based on thermodynamic principles, it is difficult to precisely control the structural perfection of either pure zinc blende or pure wurtzite. However, the bonding energy between the surface ions and surface ligands varies from 50 to 150 kJ/mol, which is extremely large compared to the CdSe core [21]. The coordination chemistry greatly influences the nucleation and growth, as well as the surface state of CdSe. In an earlier synthesis system, the effect of coordinating solvents (such as trioctylphosphine oxide) on the surface of quantum dots was much greater than that of other ligands [22,23]. To modulate the nucleation and growth of quantum dots, it is necessary to adopt a synthesis route using a non-coordinating solvent. For this purpose, Peng’s group pioneered the use of octadecene—which has a lower melting point than trioctylphosphine oxide—to develop a green and easily handled synthesis route [24]. This non-coordinating solvent reaction system has greatly promoted the development of the surface ligand chemistry of QDs. After years of research, it has been proven that the coordination between surface atoms and ligands determines the crystal phase. For instance, using cadmium phosphonate and cadmium carboxylate ligands as precursors tends to form wurtzite structures and zinc blende CdSe, respectively, while neutral ligands, such as fat amines, fatty acids, etc., have little influence [21,25].

Besides the ligand, surface passivation by constructing a core–shell structure is equally important to the fluorescence properties. QDs with an alloyed shell usually exhibit a wide emission peak (>100 meV) because the gradient interface between the core and the shell has a weak electron confinement [26,27]. Therefore, core–shells with a heterostructure are more suitable for laser application. However, it is reported that epitaxial growth of other semiconductor materials on a zinc blende CdSe core did not allow for retention of the original crystal structure, which might cause core–shell NCs with undesired optical properties. To solve this problem, the thermal-cycling technique coupled with a single precursor (TCSP) can be employed. Using this method, Nan et al. have acquired a CdSe/CdS core–shell heterostructure with a quantum yield (QY) higher than 90% [28]. The key to the TCSP technique is the removal of excess selenium (Se) before shell growth, using tributyl phosphine (TBP) as the scavenger. In fact, employment of TBP as the solvent (or scavenger) of Se powder and as the ligand of QDs has been widely reported [28], showing that the ligand chemistry is essentially important to the quality of the whole QD synthesis. However, comprehensive insight into the mechanism of TBP in QD synthesis has not been fully revealed.

The objective of this work is to understand the interaction between the ligand and core–shell structures. First, this work systematically demonstrates the process of crystal transformation under different reaction conditions in terms of the precursor concentration and TBP dosage. The structure-dependent fluorescence properties are studied by XRD, photoluminescence spectra and UV-vis spectra, TEM observation, and time-correlated single photon counting (TCSPC) technology. With precise control of the pure zinc blende CdSe core, a series of CdSe/ZnS (0.6–2.7 monolayer ZnS) and CdSe/CdS (0.8–8.1 monolayer CdS) core–shell QDs with different shell thicknesses are then prepared by a single precursor method. The influence of TBP on the growth behavior of ZnS or CdS shells is then studied by analyzing the PL spectrum and decay dynamics. With the TBP modification, a significant improvement in the coating efficiency of the ZnS or CdS shells onto the CdSe core and, hence, in the single-channel decay of emission is demonstrated.

## 2. Experiments

### 2.1. Preparation of Cadmium Stearate

First, 20 mmol of stearic acid was neutralized with equal moles of tetramethylammonium hydroxide in 200 mL of methanol. Another 50 mL of methanol solution was prepared by dissolving 10 mmol of cadmium acetate (Cd(Ac)_2_·2H_2_O). Then, the stearic acid solution was added dropwise into the Cd(Ac)_2_·2H_2_O solution, after which the white cadmium stearate (Cd(St)_2_) immediately precipitated. The mixture was kept stirring for 30 min to ensure a complete reaction. Subsequently, the white precipitate was collected through filtration and then washed three times with methanol. The final product was obtained by drying under vacuum condition.

### 2.2. Preparation of Zinc Diethyldithiocarbamate

In a typical synthesis, 15 mmol of zinc acetate dihydrate was first dissolved in 100 mL of deionized water. Another solution, prepared by dissolving 30 mmol of sodium diethyldithiocarbamate trihydrate in 60 mL of deionized water, was added dropwise into the above zinc acetate solution, with vigorous stirring. White zinc diethyldithiocarbamate (Zn(DDTC)_2_) precipitates slowly formed as the reaction progressed. The mixture was stirred for 30 min to ensure completion of the reaction. The precipitates obtained were washed three times with deionized water. Then, the separation between precipitates and water was implemented by a vacuum filtration. The final product was dried under vacuum condition.

### 2.3. Synthesis of CdSe QDs

First, 0.05 mmol (or 0.1, 0.15, 0.2, 0.4, 0.6, 0.8, and 1 mmol) of Cd(St)_2_ and 7 mL of octadecene were placed in a 50 mL three-necked flask. After degassing and nitrogen bubbling twice (10 min for each time), the mixture was heated to 250 °C. In a separate vial, TBP=Se stock solution was prepared by dissolving 0.1 mmol of Se powder into 0.2 mmol (~50 μL) of TBP and 0.95 mL of octadecene (ODE). This stock solution was swiftly injected into the hot solution so as to rapidly nucleate CdSe NCs. Needle-tip aliquots were taken for UV−vis and PL measurements to monitor the growth of the NCs. When the NCs reached the target size, the reaction solution was cooled to room temperature.

### 2.4. Synthesis of CdSe/ZnS Core/Shell Nanocrystals

To prepare a precursor solution, 0.5 mmol of Zn(DDTC)_2_ was first dissolved ultrasonically in a mixture of 2 mL of hexadecane, 2.9 mL of n-octylamine, and 0.1 mL of oleylamine. Next, the precursor solution, at various doses, and 3 mL of oleylamine were added to the cooled nuclear solution. After degassing and nitrogen bubbling twice, the mixture was heated to 100 °C. At this temperature, 75−500 μL of the TBP was subsequently injected (according to a molar ratio of TBP/*X*(DDTC)_2_ = 10, *X* = Zn or Cd) into the solution. Finally, the temperature was increased to 140 °C (ZnS) or 160 °C (CdS) for 30 min for epitaxial growth of the shell layer. The prepared colloidal QDs were precipitated by adding excess ethanol and by centrifuging at 8000 rpm for 5 min. The supernatant liquid was decanted, and the precipitation was redispersed in n-hexane for further analysis.

### 2.5. Characterizations

Structural changes of crystals were measured using an X-ray diffractometer (Bruker D8 advance, Karlsruhe, Germany). Transmission electron microscope (TEM, JEOL JEM-2100F, Tokyo, Japan) operated at an accelerating voltage of 200 kV was used to obtain high-resolution TME images. The particle diameter was measured by an analysis software (Nano Measurer 1.2, Jie Xu, Changzhou, China). More than 200 particles in TEM images were included in the statistics. The accumulation normal distribution was analyzed based on Gaussian fitting, by which the average diameter was calculated. Photoluminescence (PL) spectra were recorded with a Lambda LS55 (Perkin Elmer, Waltham, MA, USA), and absorption spectra of the films were measured by a Lambda 750 UV/VIS/NIR spectrophotometer (Perkin Elmer, Waltham, MA, USA). PL decay curves were collected at room temperature with a time-correlated single-photon counting (TCSPC) spectrofluorometer. QYs of QDs were measured by a FLS980 fluorescence spectrophotometer (Edinburgh Instrument, Livingston, UK). The components of the nanocrystals were characterized by energy disperse spectroscopy (EDS).

## 3. Results and Discussion

### 3.1. Effect of Precursor Concentration

**Figure 1:**
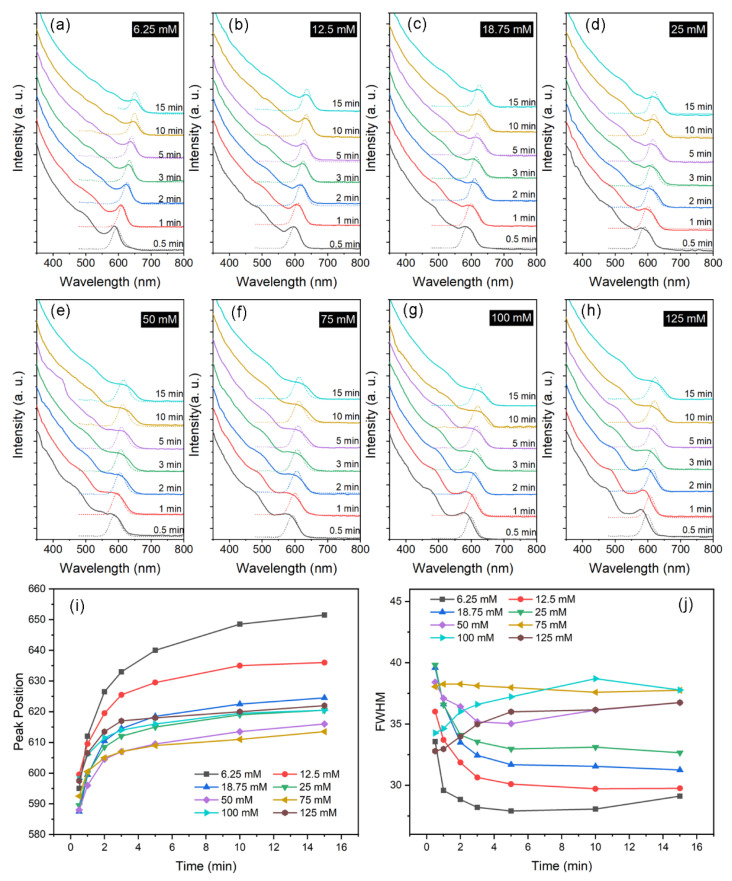
(**a**–**h**) PL and UV-vis spectra as a function of precursor concentration. Evolutions of (**i**) PL peak and (**j**) FWHM with reaction time.

The precursor concentration varies from 6.25 mM to 125 mM, which is achieved by simultaneously increasing the molarity of Cd, Se, and TBP. The PL and UV-vis spectra are shown in Figure 1a–h. Due to the quantum confinement effect, QDs exhibit different absorption capacities for light, thus showing different absorption peaks. Each absorption peak corresponds to an exciton level. The exciton peak becomes less obvious, and the absorption becomes a continuous absorption band, as the absorption wavelength moves to the short wavelength region. Therefore, the first and second exciton absorption peaks in the long wavelength range offer the most useful information on the optical properties of QDs. The narrow exciton absorption peak indicates a narrow size distribution of QDs. It can be found in Figure 1i that a higher precursor concentration produces NCs with a shorter first exciton absorption or PL peak wavelength. This means that fast nucleation of a large number of seeds leads to a larger number of particles with a smaller final size. Half height and full width (FWHM) of the PL peak becomes narrow as reaction time progresses (Figure 1j). Size focusing is supposed to occur during growth, which decreases the initial size dispersion of the NCs. The above observation shows good accordance with the LaMer model [29], which links the nucleation and growth of colloidal particles in a homogeneous solution. However, when the precursor concentration is higher than 0.075 M, a different growth behavior emerges. That is, the nucleation size becomes larger and products show a broadening size distribution.

**Figure 2:**
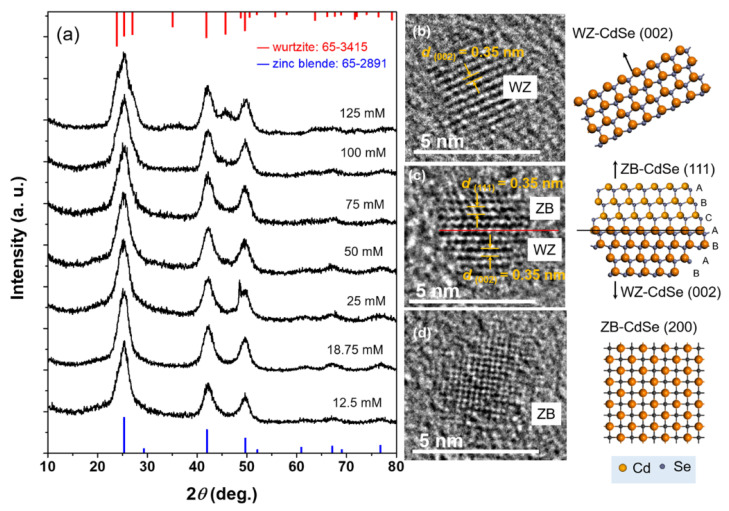
(**a**) XRD patterns of CdSe QDs prepared at different precursor concentrations. TEM images of CdSe NCs with (**b**) wurtzite structure, (**c**) wurtzite/zinc blende hybrid structure, and (**d**) zinc blende structure.

To investigate the growth mechanism, XRD patterns of the final NCs are plotted in Figure 2a. Diffraction peaks at 25.5°, 29.5°, 42.2°, and 49.9° are assigned to the (111), (200), (220), and (311) planes of a standard zinc-blende CdSe (JCPDF 65-2891) [30]. When the precursor concentration is lower than 50 mM, the NCs display the same zinc blende structure. Increasing the precursor concentration to 75 mM results in a different XRD pattern. The (111) peak of the zinc blende structure at about 25.5° evolved into a broad envelope due to the overlap of the (100), (002), and (101) Bragg peaks of the wurtzite structure. A new peak also emerges at ~45.5°, indicating a hybrid structure composed of zinc blende (ZB) and wurtzite (WZ) components. When the precursor concentration is as high as 125 mM, the wurtzite CdSe becomes the main component. TEM images are shown in Figure 2b–d, so as to directly offer structural information at an atomic level. From the images, three NCs with different crystal types were verified, including WZ-CdSe (Figure 2b), WZ-ZB hybrid CdSe (Figure 2c), and ZB-CdSe (Figure 2d). Therefore, the precursor concentration plays an important role in the crystalline structure.

**Figure 3:**
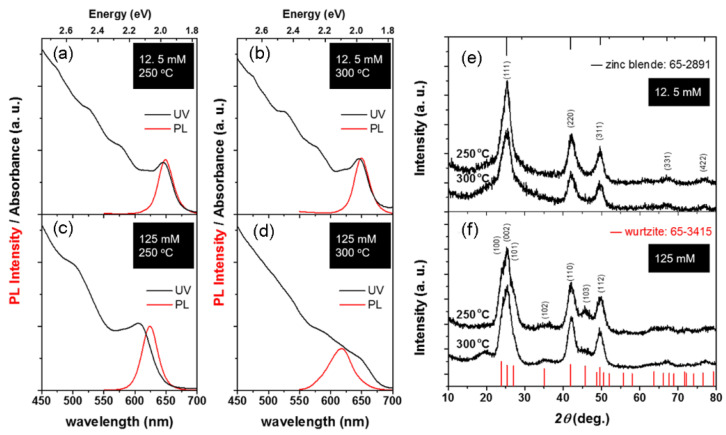
(**a**–**d**) PL and UV-vis spectra of CdSe NCs prepared under different synthesis conditions. (**e**,**f**) The corresponding XRD patterns of CdSe NCs.

An early work reported that the phase transition temperature from zinc blende (room temperature stable) to wurtzite (high temperature stable) for bulk CdSe is about 100 °C [31]. Maintaining crystal structure at different synthesis temperatures is still a challenge. Figure 3 shows the PL and UV-vis spectra, and XRD patterns of CdSe NCs prepared at 250 and 300 °C, respectively. It can be seen that when the precursor concentration is fixed, their fluorescence properties and structure are maintained despite the change in temperature. It should be noticed that the reaction proceeding at high precursor concentration and at high temperature (300 °C) easily causes fast growth of the CdSe core and leads to precipitated NCs in solution. This can be verified by comparing the PL and UV-vis spectra in Figure 3c,d, where the redshift and the broadening of the first exciton absorption peak can be observed. This temperature-independent structural feature means that there is a wide range of thermodynamic tolerance for developing facile and effective pathways to precisely control the fluorescence properties.

### 3.2. Influence of TBP

**Figure 4:**
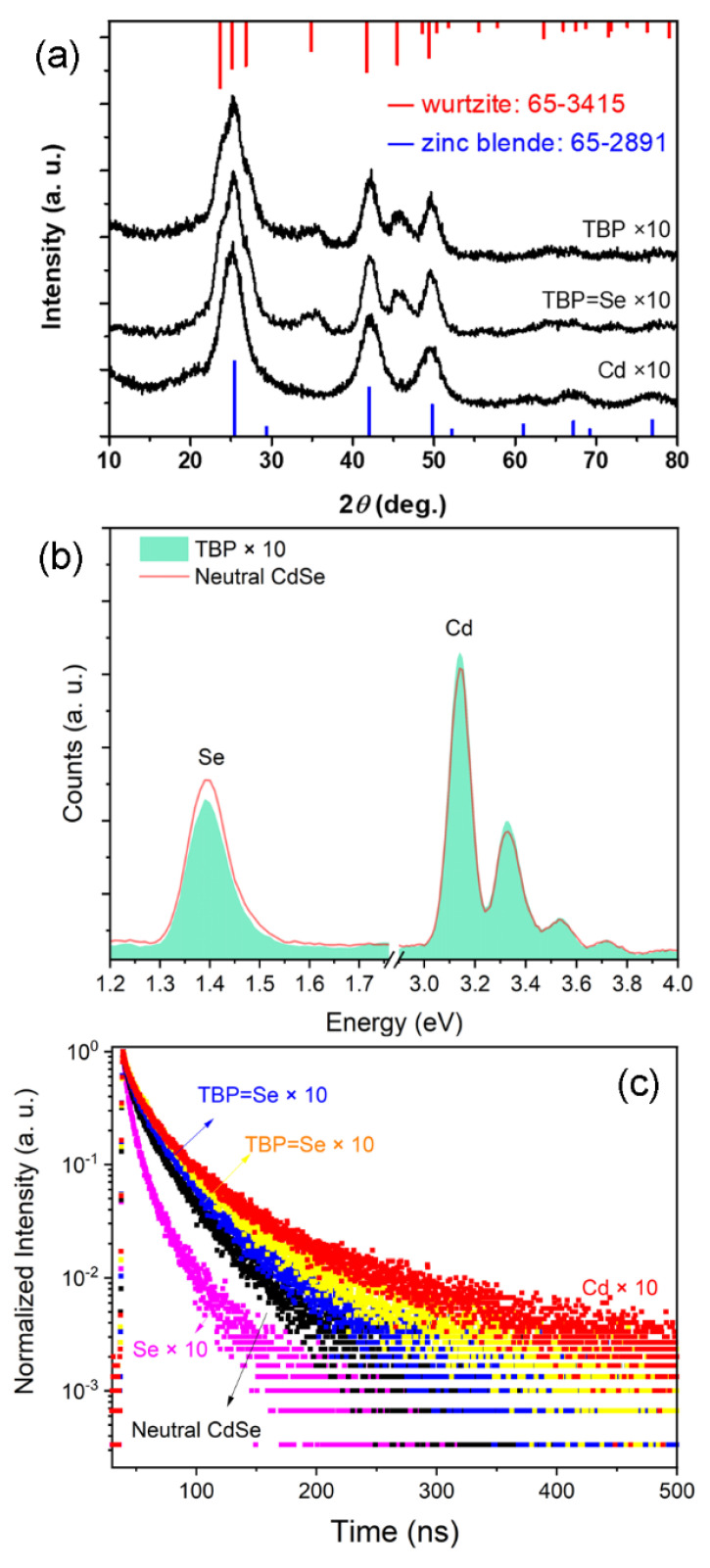
(**a**) XRD profiles of CdSe NCs. (**b**) EDS spectra of CdSe NCs. (**c**) PL decay curves.

TBP has been widely used in the synthesis of CdSe and other types of NCs [32,33,34]. However, there is uncertainty in terms of its influence on the crystal structure. To clarify the effect of TBP, a set of samples was compared. The first sample was prepared with a 10-fold excess of Cd (labelled as Cd × 10). The second sample was prepared by increasing the dosage of TBP=Se by 10 (labelled as TBP=Se × 10). The third sample was prepared by increasing the usage of TBP by 10, while the dosages of Cd and Se were kept constant (labelled as TBP × 10). Their XRD patterns are shown in Figure 4a. For the first sample, the resulting nanocrystals exhibit a pure zinc blende structure, which is in line with that of the pristine CdSe. Therefore, it can be concluded that a Cd-rich environment only slightly influences the structural changes of CdSe. Both the second and third samples exhibit the same wurtzite-type structure, confirming the dominant role of TBP in structural change. The energy-dispersive spectrometer (EDS) was used to analyze elemental proportions of QDs. As shown in Figure 4b, the atomic ratio of Se/Cd in CdSe QDs prepared by the conventional method is higher than that prepared with excessive TBP. Specifically, with the addition of TBP, the atomic ratio of the Se element in purified core QDs significantly decreases from 41.7% to 35.1%. For such small particles, surface atoms account for most of the total number of atoms. The change in the total number of atoms is almost equal to the change in the number of surface atoms. This indicates that the TBP in the reaction system helps to remove Se ions that are on the QD surface. To verify this hypothesis, PL decay curves are shown in Figure 4c. Compared to QDs with a neutral surface, it can be found that the decay lifetime is lengthened or shortened with a Cd-rich or Se-rich surface, respectively. Herein, when the dosage of TBP is increased tenfold, a fast decay component appears in the dynamics curve. This result strongly verifies a depleted state of the Se ion on QD surface.

### 3.3. CdSe/ZnS Core–Shell Nanocrystal

**Figure 5:**
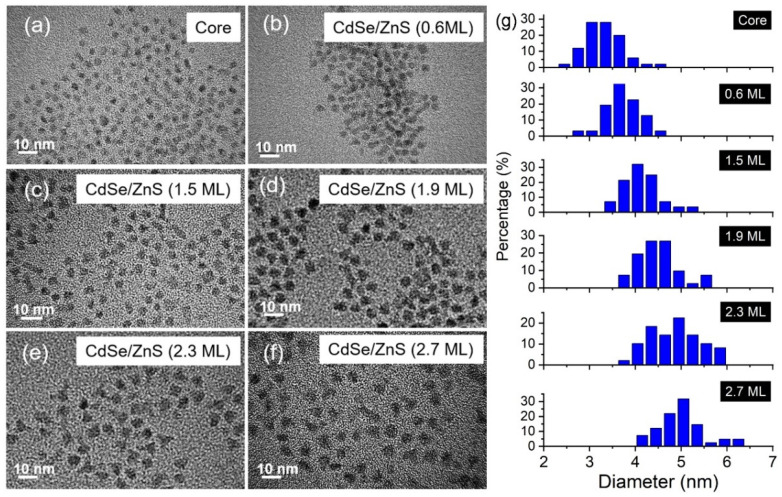
TEM images of (**a**) CdSe core and (**b**–**f**) CdSe/ZnS core–shell NCs with different ZnS thicknesses. (**g**) Statistical distribution histograms.

**Figure 6:**
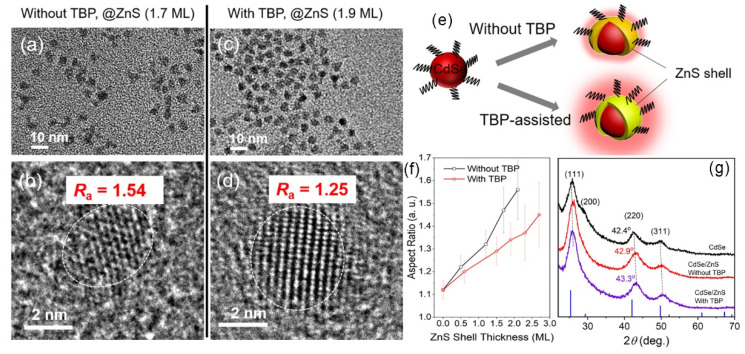
High-resolution TEM images of CdSe/ZnS QDs (**a**,**b**) without or (**c**,**d**) with TBP. (**e**) Schematic diagram of the influence of TBP on shell growth. (**f**) Aspect ratio as a function of ZnS shell thickness. (**g**) XRD patterns of CdSe/ZnS QDs.

Given that the average thickness of one monolayer (ML) of ZnS is ~0.31 nm [35,36], the number of coating MLs can be estimated by counting the particle size on TEM measurements. As shown in Figure 5a, the average diameter of the CdSe core is 3.47 nm. With the reaction of Zn(DDTC)_2_ at a moderate temperature, CdSe/ZnS NCs with a heteroepitaxial core–shell structure were successfully synthesized. Their morphologies are shown in Figure 5b–f. Particle size statistics are summarized in Figure 5g. The thicknesses of the ZnS shell were 0.6, 1.5, 1.9, 2.3, and 2.7 ML, corresponding to dosages of 0.03, 0.05, 0.08, 0.13, and 0.20 mmol of Zn(DDTC)_2_, respectively. Note that the particle size is reduced by 0.1–0.3 ML when TBP is absent. For instance, when the reaction proceeds with 0.08 mmol of Zn(DDTC)_2_ without TBP, the resulting thickness of ZnS is only 1.7 ML (see comparison in Figure 6a–d). In addition, the particle shape changes in different reaction systems. Most of the NCs in Figure 6a are non-spherical, indicating the anisotropic growth of the ZnS shell. Herein, the aspect ratio parameter (*R*_a_, defined as a ratio of the horizontal length and the vertical length of a nanocrystal) is introduced to evaluate the anisotropic growth. Without TBP, the *R*_a_ of the nanocrystal is as large as 1.54. By contrast, the resulting core–shell QDs with TBP addition are nearly spherical (Figure 6c), and the size distribution becomes narrower (with an *R*_a_ of 1.25). Therefore, TBP effectively suppresses the anisotropy of epitaxial growth and improves the coating efficiency of ZnS, as illustrated in Figure 6e. According to the TEM observations, the *R*_a_ of the nanocrystal as a function of the ZnS shell thickness is plotted in Figure 6f. To further confirm this improved coating efficiency, XRD profiles are presented in Figure 6g. Four diffraction peaks, including CdSe (111), (200), (220), and (311), change with the reaction conditions. Taking the CdSe core as a reference, the heteroepitaxial growth of ZnS causes the diffraction peak to shift to a larger angle. For the CdSe/ZnS synthesized without TBP, its (220) diffraction peak is located at 42.9°, while its counterpart with TBP addition is centered at a larger angle of 43.3°. This means that TBP increases the growth rate of ZnS, which is in good accordance with the TEM observation.

**Figure 7:**
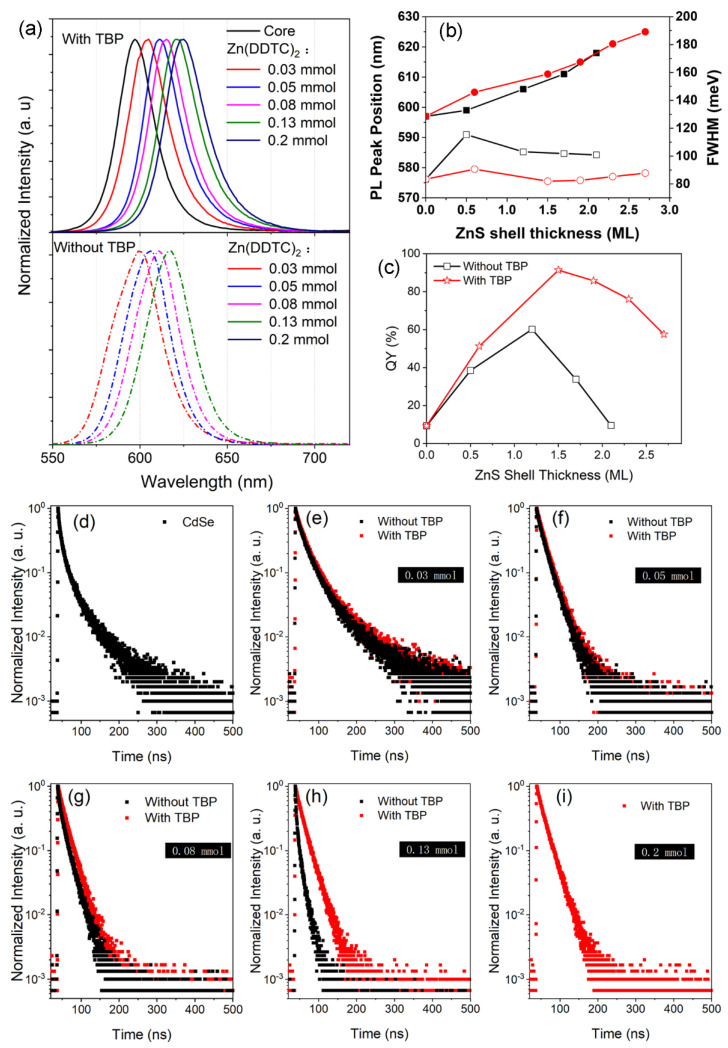
(**a**) Evolution of PL spectra with different ZnS shell thicknesses. (**b**) Fluorescence properties in terms of peak position and FWHM of the emission. (**c**) QY value as a function of CdS shell thickness. (**d**) PL decays of CdSe QDs. (**e**–**i**) PL decays of CdSe/ZnS QDs with different shell thicknesses.

The size-dependent PL emissions are presented in Figure 7a. The emission wavelengths and the FWHM of PL spectra as a function of ZnS thickness are plotted in Figure 7b. The heteroepitaxial structure of ZnS onto CdSe leads to a redshift of the emission, from 597 to 625 nm. This redshift is noticeably larger than that of core–shell NCs prepared under the same conditions except for the absence of TBP. This indicates faster ZnS growth, which is in line with the results from TEM and XRD measurements. In the conventional synthesis (without TBP), the peak FWHM increases from 84 meV to above 100 meV as reaction time progresses. By contrast, the introduction of TBP helps to sustain a narrow FWHM at a range of 82–90 meV, implying an improvement in the fluorescence properties as reaction time progresses. In addition, the resulting NCs possess a longer emission wavelength but a higher PL QY (Figure 6b) in the TBP-containing system. For example, the QY for NCs with a 1.2 ML ZnS shell is only 60.1% at an emission wavelength of 606 nm, but the comparative sample (with 1.5 ML-thick ZnS) dramatically increases to 91.4% at 611 nm.

From Figure 7c, it can be found that a shell thickness larger than 1.9 ML deteriorates the PL emission. To investigate the quenching mechanism, PL decay measurements were performed. The decay curves and fitting results are shown in Figure 7d–i and Table 1, respectively. The decay dynamics of the CdSe core (Figure 7a) has a dual-channel characteristic. The corresponding lifetime components are assigned to the surface-defect state transition (*τ*_1_) and the intrinsic transition (*τ*_2_). In the initial coating stage, i.e., when the ZnS shell thickness is less than 1 ML, the average lifetime (*τ*_ave_) increases. This stems from cationic (Zn^2+^) adsorption onto the QD surface, as proved by Peng’s group [37]. The short lifetime component always exists when TBP is absent, suggesting that surface defects of QDs are not completely passivated. However, the QD exhibits single-channel decay dynamics after TBP treatment, implying the elimination of radiation from surface states. Note that the average lifetime of QDs decreases with the increase in ZnS thickness, while that of QDs synthesized with TBP is nearly unchanged. This means that the addition of TBP, which acts as the ligand on the QD surface, greatly enhances the stability of the core–shell structure.

### 3.4. CdSe/CdS Core–Shell Nanocrystal

**Figure 8:**
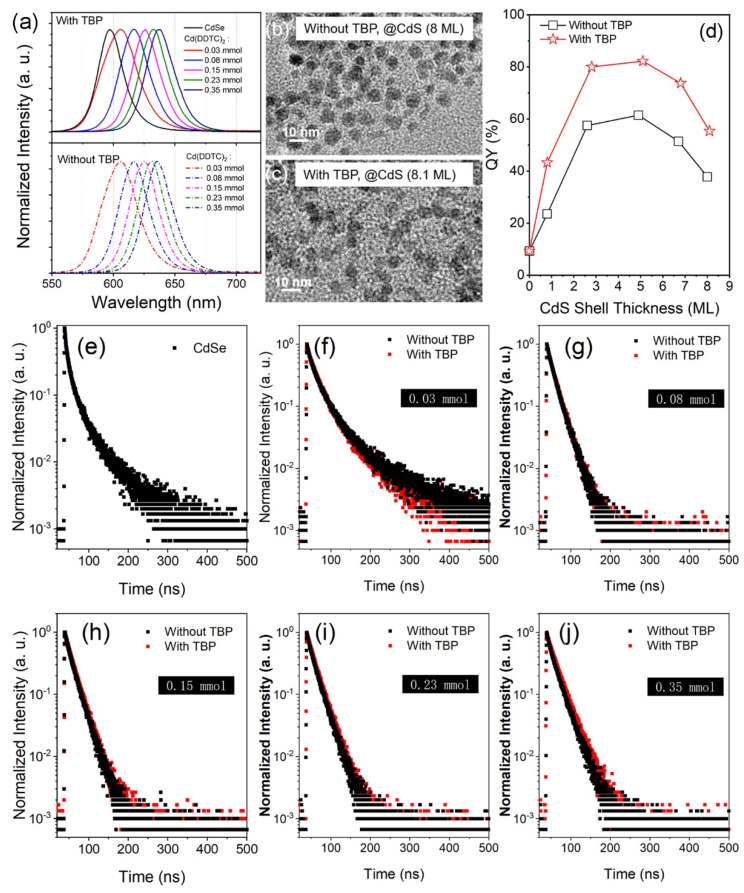
(**a**) Evolution of PL spectra with different CdS shell thicknesses. (**b**,**c**) TEM images of the CdSe/CdS QDs. (**d**) QY value as a function of CdS shell thickness. (**e**–**j**) PL decays of CdSe/CdS QDs with different shell thicknesses.

The CdSe/CdS core–shell structure NCs were synthesized via a similar route. The PL spectra are shown in Figure 8a. The heteroepitaxial structure of ZnS on CdSe leads to a redshift of the emission from 597 to 638 nm. In this case, the presence of TBP in the reaction system has less impact on the PL emission. The shape and the size of the NCs are almost the same (Figure 8b,c). However, the TBP still plays an important role in the fluorescence mechanism. As can be seen in Figure 8d, the QY value is much higher than that of QDs synthesized without TBP. Decay curves and fitting parameters are shown in Figure 8e–j and Table 2, respectively. Without TBP, the CdSe/CdS exhibits single-channel decay dynamics only when CdS thickness is at a range of 4.9–6.7 ML. Moreover, due to the nonradiative recombination, its average lifetime is shorter than that of the QDs synthesized with TBP. Therefore, this case further proves the significance of TBP in improving fluorescence properties and in enhancing the stability of core–shell structure.

## 4. Conclusions

In summary, CdSe and CdSe/ZnS NCs with tunable crystalline structure and fluorescence properties have been successfully synthesized. Reaction conditions, in terms of precursor concentration and TBP dosage, have shown their influence on the nucleation and growth of the CdSe core. When the precursor concentration (with TBP) is higher than 75 mM, the structure of CdSe tends to transform from the zinc blende to the wurtzite structure. Adding an appropriate amount of TBP in the single precursor coating process can help to inhibit the anisotropy of epitaxial growth and thus improve the coating efficiency. Based on the precise control of the pure zinc blende CdSe core, a series of CdSe/ZnS (0.6–2.7 monolayer ZnS) and CdSe/CdS (0.8–8.1 monolayer CdS) core–shell QDs with different shell thicknesses were then prepared. The improved QDs show single-channel decay dynamics, and the QY value for CdSe/ZnS (CdSe/CdS) heterogeneous core–shell QDs dramatically increases from 60.1% (61.4%) to 91.4% (82.2%). The excellent fluorescent performance of QDs with heterogeneous core–shell structure presented in this work may greatly promote their application in lasers and other fields.

## Figures and Tables

**Table 1:** Lifetime and fractional contribution of different decay channels of CdSe/ZnS QDs.

Structure	Shell Thickness (ML)	*τ*_1_ (ns)	*B*_1_	*τ*_2_ (ns)	*B*_2_	*τ*_ave_ (ns)	*χ* ^2^
CdSe	0	9.7	49.9	49.2	50.1	42.7	1.04
CdSe/ZnS (Without TBP)	0.5	22.9	68.3	84.7	31.7	62.0	1.10
	1.2	16.3	64.8	32.8	35.2	24.9	1.05
	1.7	6.9	22.2	20.4	77.8	19.2	1.01
	2.1	3.2	39.1	13.5	60.9	12.1	1.08
CdSe/ZnS (With TBP)	0.6	24.8	69.2	94.3	30.8	68.5	1.16
	1.5	—	—	22.7	100	22.7	1.21
	1.9	—	—	20.2	100	20.2	1.24
	2.3	—	—	19.9	100	19.9	1.05
	2.7	—	—	20.9	100	20.9	1.28

**Table 2:** Lifetime and fractional contribution of different decay channels of CdSe/CdS QDs.

Structure	Shell Thickness (ML)	*τ*_1_ (ns)	*B*_1_	*τ*_2_ (ns)	*B*_2_	*τ*_ave_ (ns)	*χ* ^2^
CdSe	0	9.7	48.8	49.2	512	42.6	1.12
CdSe/CdS (Without TBP)	0.8	22.9	65.0	96.8	35.0	74.2	1.27
	2.6	12.3	34.7	23.5	65.3	21.1	1.01
	4.9	—	—	19.6	100.0	19.6	1.15
	6.7	—	—	20.2	100.0	20.2	1.25
	8	9.8	15.4	24.5	84.6	23.5	1.06
CdSe/CdS (With TBP)	0.8	23.5	68.9	86.6	31.1	68.8	1.07
	2.8	—	—	19.6	100.0	21.0	1.10
	5.1	—	—	20.5	100.0	20.5	1.05
	6.8	—	—	21.4	100.0	21.37	1.07
	8.1	—	—	22.7	100.0	22.7	1.19

## Data Availability

The data presented in this study are available in this paper.
